# ‘Joining a group was inspiring’: a qualitative study of service users’ experiences of yoga on social prescription

**DOI:** 10.1186/s12906-022-03514-3

**Published:** 2022-03-14

**Authors:** Anna Cheshire, Rebecca Richards, Tina Cartwright

**Affiliations:** 1 School of Social Sciences, University of Westminster, 115 New Cavendish Street, London, W1W 6UW UK; 2 MRC Epidemiology Unit, University of Cambridge, Cambridge, UK

**Keywords:** Yoga, Social prescription, Patient, Qualitative analysis, Interview, Mental health, Primary care, National Health Service, Complex intervention

## Abstract

**Background:**

Yoga is becoming an increasingly popular holistic approach in the West to manage long-term health conditions. This study presents the evaluation of a pilot yoga intervention, Yoga4Health, that was developed for the NHS to be socially prescribed to patients at risk of developing specific health conditions (risk factors for cardiovascular disease, pre-diabetes, anxiety/depression or experiencing social isolation). The aim of this qualitative study was to explore service users’ experiences of Yoga4Health and the acceptability of the programme.

**Methods:**

Qualitative data were collected from three sources: 1. Open-ended questions on questionnaires completed by services users at three different time-points (baseline, post intervention and 3 months); 2. Interviews and focus groups with a subset of participants (*n* = 22); 3. interviews with yoga teachers delivering Yoga4Health (*n* = 7). Each data source was analysed thematically, then findings were combined.

**Results:**

Of participants completing baseline questionnaires (*n* = 240), 82.5% were female, 50% White, with a mean age of 53 (range 23–82) years. Baseline questionnaires revealed key motivations to attend Yoga4Health were to improve psychological and physical health, and believing Yoga4Health would be accessible for people with their health condition. Post-intervention, participants reported a range of benefits across psychological, physical and social domains from Yoga4Health. Increased confidence in self-management of health was also reported, and a number of participants described making positive lifestyle changes after attending the programme. Unanticipated benefits of yoga emerged for participants, such as enjoyment and social connectedness, which facilitated ongoing attendance and practice. Also key to facilitating practice (during and after the intervention) were suitability of the classes for those with health conditions, practising with a group and qualities of the yoga teacher. Home practice was supported by course materials (manual, videos), as well as the teaching of techniques for everyday application that offered immediate benefits, such as breathing practices. Follow-up questionnaires revealed a key challenge was continuation of practice once the intervention had finished, with the structure of a class important in supporting practice.

**Conclusions:**

Yoga4Health was a highly acceptable intervention to services users, which brought a range of biopsychosocial improvements, suggesting yoga is an appropriate intervention to offer on social prescription.

**Supplementary Information:**

The online version contains supplementary material available at 10.1186/s12906-022-03514-3.

## Background

Social prescribing is a means of enabling general practitioners and other primary healthcare professionals to formally refer patients to a range of non-clinical community services. Its aim is to support people in a holistic way, recognising that health is influenced by a range of social, economic and environmental factors [1]. Pioneered in the UK, social prescribing has recently become widely available in general practices across the country [2]. Often patients are initially referred to a link worker where they learn about what community services are available, and are able to co-produce their own ‘social prescription’ according to their individual needs, for example, volunteering, arts activities, sports, and singing. However, many National Health Service (NHS) localities are also now offering or considering implementing their own social prescribing programmes in addition to those offered by the community. Yoga is one activity that may be a suitable candidate for this new social prescribing pathway given the current policy focus on holistic care and illness prevention [3, 4].

Yoga is a holistic mind-body practice originating from India but increasingly recognized as a therapeutic intervention to prevent and manage health conditions [5]. It has also become increasingly popular in the West as an accessible approach to support health and wellbeing [6, 7]. There are a variety of styles of yoga, however most incorporate physical postures (asana), breathing techniques (pranayama), meditation (dhyana) and relaxation. Both cross-sectional population surveys and clinical trials suggest that yoga has physiological and psychological effects on health, as well as being associated with positive health behaviours such as higher levels of physical activity [6, 8–12]. Of particular interest to the current study is evidence for the effectiveness of yoga for cardiovascular disease, type 2 diabetes, mental health and social isolation.

Systematic reviews have reported the potential effects of yoga interventions on cardiovascular risk factors in the general population and high risk groups (e.g. those with hypertension, metabolic syndrome, and type 2 diabetes) [13, 14]. Compared to control groups, yoga groups were found to have significant improvements on a range of physiological measures (e.g. diastolic blood pressure, HDL cholesterol) [14], waist circumference, body mass index and quality of life [13–15]. Additionally, qualitative research has found yoga to have ancillary benefits for populations at risk of cardiovascular disease, including reduced stress and anxiety, better sleep and improved diet [16]. Similar findings have been reported for the effects of yoga for adults with type 2 diabetes [17]; compared to controls, yoga showed significant improvements in markers of glycemic control (e.g. reductions in HbA1c) and body composition [18, 19]. Furthermore, a recent study of a community-based ten-day yoga intervention reported a significant reduction in fasting blood glucose for adults with a diagnosis of pre-diabetes, which suggests yoga may prevent worsening glycaemic control for adults in the pre-diabetic range [20].

Yoga has been found to have robust effects on poor mental wellbeing – notably, by decreasing anxiety and depression [21, 22], as well as physiological markers of stress, such as blood pressure, heart rate, and cortisol [11, 23]. For example, a meta-analysis of 12 randomised control trials of yoga interventions for patients with depressive disorders and elevated levels of depression found reductions in severity of depression compared to receiving standard care only [21]. Yoga interventions have also been shown to reduce perceived levels of stress both in healthy adults and ageing populations [24, 25]. For non-clinical populations, the effects of yoga on positive mental well-being (e.g. satisfaction with life) have been shown to be similar to those of physical activity [26].

As well as offering physical and mental health benefits, yoga has been associated with increased social connectedness and has been advocated for populations at risk of social isolation, such as the elderly, or those who are bereaved or have depression [27, 28]. A small but growing number of studies have investigated the effects of yoga on social connectedness, in both the general population and high-risk groups. Findings suggest that yoga creates a sense of community for those who practice it, both practically and emotionally, as participants report meeting new friends and feeling like they belong [27, 28]. Additionally, yoga may improve existing interpersonal relationships by changing attitudes and perspectives, including improving patience, kindness or self-awareness [27, 29].

Yoga interventions, which typically include weekly or bi-weekly instructor led group classes for 8–12 weeks, have been found to be acceptable for a range of populations, including those with depression [30], type 2 diabetes [31, 32], and at risk of cardiovascular disease [16]. In addition, the NHS describes yoga as a safe and effective physical activity for people with a range of chronic conditions [33]. Thus, the growing evidence of the effectiveness of yoga for health conditions, the social nature of yoga and its acceptability, have made it an activity that is likely suitable for social prescribing [3].

This study reports on qualitative evaluation findings exploring participants’ experiences of a pilot yoga intervention, called Yoga4Health. Yoga4Health was developed for the NHS to be socially prescribed to patients at risk of developing specific health conditions, including those at risk of cardiovascular disease or pre-diabetes, with mild to moderate anxiety or depression, or who are socially isolated. It is important to conduct early phase qualitative studies of interventions to assess acceptability to services users, optimise their experiences and support uptake upon implementation in the community [34]. Whilst there is good evidence that yoga will be helpful in this context, some practitioners may experience barriers to engagement. For example, classes may be too challenging for those with more limited physical abilities [30, 35], at inconvenient times [31, 35], or perceived to be not for “people who look like me” [35, 36]. Therefore, the aim of this study was to assess the acceptability and feasibility of the intervention, by exploring service users’ experiences of Yoga4Health, and facilitators and barriers to participation and continued yoga practice.

## Methods

### Study design

Frameworks to guide the development of complex interventions in healthcare advocate a mixed-methods approach to evaluate the feasibility and acceptability of an intervention during its pilot phase [34, 37]. Here a nested qualitative design was used, where participants completed questionnaires of validated outcome measures (and open-ended questions) at three different time-points; a subset of participants completed individual interviews or participated in focus groups. In addition, yoga teachers involved in delivering Yoga4Health were interviewed about their experiences and perceptions of the intervention. This study reports on the qualitative data only, results from the quantitative data are reported elsewhere (Cartwright T, Richards R, Edwards A, Cheshire A: The impact of a yoga social prescribing programme on patient-reported outcomes, in preparation). Qualitative methods capture personal experiences through the collection of contextual and in-depth data [38], here we triangulated data from three sources: open-text responses included within the study questionnaires enabled us to capture the experiences of the whole sample. Interviews and focus groups provided a complementary combination of data collection methods, with focus groups supporting group discussion and debate [39] and interviews allowing a deeper exploration of service users’ and yoga teachers views and experiences. Additionally, interviews provided an option for participation for those who are unable to attend focus groups.

### Participants and recruitment

In total, 365 service users were booked onto the Yoga4Health programme and 279 (76%) attended at least one session with 240 (86%) completing a baseline questionnaire. The programme was advertised with leaflets through general practice, community organisations (e.g. Healthy Hearts) and outreach. The main method of referral was from mass text message campaigns inviting targeted patients who met the eligibility criteria, which were sent by the provider (Thrive Tribe) from general practices across the clinical commission group area. Patients either self-referred to the programme or were referred by their general practitioner. Each interested patient received a telephone call to discuss the intervention and evaluation and to book a place on a course if they met the intervention inclusion criteria, which included: mild to moderate anxiety or depression (self-reported), social isolation (self-reported), risk factors for cardiovascular disease (Q risk over 10%) or pre-diabetes, (see Additional file 1 for exclusion criteria). All patients who were booked onto a course were invited to take part in the evaluation.

Purposive sampling was used to select a subsample of participants (*n* = 22) to invite to interview/focus groups [40]. To allow a range of views to emerge, participants were sampled along dimensions of ethnicity, age and number of sessions completed. Seven participants from the initial Yoga4Health courses were invited to take part in an interview, five were available and were interviewed. Additionally, three focus groups (with three to eight participants) were conducted with participants from subsequent courses; all of those invited agreed to take part. Seven (out of 11) yoga teachers who delivered Yoga4Health were invited and agreed to be interviewed, including two involved in programme design. All teachers who had delivered a Yoga4Health course at the time of interviews were invited.

### Intervention

The Yoga4Health programme is a ten-week manualised group yoga course specifically designed to provide a safe and supportive environment for service users, irrespective of yoga experience or ability. It was designed by yoga teachers and therapists with experience of working with people with mental and physical health conditions (https://yogainhealthcarealliance.com/yoga-for-health-program/). To enable individualised attention and modifications, the programme was designed for classes of a maximum of 15 service users. Each weekly session was 2 h long, comprising of: i) 5 min of psycho-education on a different theme each week which was then woven throughout the class (e.g. the importance of deep breathing techniques for relaxation), ii) approximately 1 h of asana practice (yoga postures), iii) 5–10 min of a formal practice of breathing, iv) a relaxation-based activity, and v) a group discussion involving experiences of the practice and any questions (15 min). In order to support the weekly sessions and maximise the impact of yoga practice on health, regular home practice was encouraged and supported by a range of materials, including a written course manual, videos and handouts. Service users were offered four variations of home practice each week, to provide a more individualised approach (choice of mat or chair and short or long session). The programme was initially delivered by the founders of the programme and subsequently by experienced and qualified yoga practitioners (regulated by their professional body) who underwent specialised training to deliver the course. A total of 22 yoga courses took place between February 2018 and May 2019, in community venues. At the end of the programme, information was provided on inexpensive local yoga classes to support further engagement in physical activity.

### Data collection

Each evaluation questionnaire (baseline, post-intervention and 3-month follow-up) included free text open-ended questions. At baseline, participants were asked what they hoped to get out of attending the yoga course. Post-intervention participants were asked about: 1) any benefits they felt they had gained from practising yoga, 2) any changes to their lifestyle made as a result of attending the yoga classes, 3) their overall experience of the yoga course, such as what they enjoyed, found challenging or any improvements or changes they would like to see, and 4) any further comments they had about the course. At 3-month follow-up, participants were asked 1) whether there was anything that made it difficult to practice yoga after the sessions finished, 2) whether there was anything that helped them to continue to practice yoga, 3) any benefits that they were currently experiencing from practising yoga, and 4) any further comments about the course.

Evaluation participants who had expressed an interest in taking part in an interview (via their consent form) were sent an email invitation. All interviews and focus groups were conducted face-to-face. Interviews took place at the participant’s home or at the University, focus groups in community centres where the yoga classes had taken place. They were conducted by RR (Research Associate, female, PhD) who was not known to the participants. RR has experience of conducting qualitative interviews for health research with patient groups and practicing reflexivity to support the data collection and analysis process [41]. While RR has an interest in health and well-being, she has not practiced yoga so did not have personal experience to relate to participants' narratives. Some participants may have had preconceived ideas about University researchers and/or may have been conscious that the interviewer was in contact with their yoga teachers. RR was aware that this might influence participants’ trust and openness during data collection, so made every effort to build rapport prior to and during data collection, such as adopting a friendly and transparent approach. RR assured participants that the interviews were confidential, that their views and opinions would not be discussed with their yoga teachers and any data published as a result of this study would be anonymised and could not be linked back to them in any way.

A semi-structured approach to data collection was adopted. Topics covered included experiences of yoga4health, and perceptions of acceptability, usefulness, facilitators of/barriers to the programme, benefits/disadvantages of the programme and home practice (see Additional file 2 for interview schedule). The topic guide for the focus group was based on the interview guide but was simplified into fewer questions (see Additional file 3 for focus group questions). Interviews lasted between 22 and 39 min (mean duration 30 min); focus groups lasted 53 to 63 min (mean duration 56 min). Interview topics for yoga teachers included experiences of delivering Yoga4Health classes and perceived usefulness for participants (see Additional file 4 for interview questions). Interviews lasted between 24 and 52 min. Data saturation was reached with this data collection. All interviews and focus groups were audio-recorded and transcribed verbatim by a professional transcriber who signed a confidentiality agreement. Participant transcript and theme checking were not used in this study. The Consolidated criteria for reporting qualitative research (COREQ) were followed to enhance the rigour and transparency of the qualitative research [42].

### Qualitative analysis

All qualitative data from questionnaire free responses, interviews and focus groups were analysed inductively, using thematic analysis [43]. Questionnaire free responses were organised using Excel, interview and focus group data were coded in NVivo [44]. Initially, free-response questions were analysed individually, with RR immersing herself in the data by reading the responses several times. An initial list of codes were assigned to responses for each question, which were then organised into themes and subthemes. Codes and themes were then refined several times to create a coherent list of final codes and themes for each question. Data from interviews (patient and yoga teacher) and focus groups were analysed initially by RR then together AC and TC built on and extended the analysis; meanings and patterns throughout the data set were explored, which led to the generation of codes for the dataset. Draft themes, their relationships and how to order/present them were discussed between all authors. The analysis of the open-ended responses from questionnaires was then integrated with the interview and focus group analysis. The themes and codes of the questionnaire data were therefore considered in relation to the themes and codes of the interview and focus group data, and vice versa. This process was discussed collaboratively and final themes agreed amongst the research team.

## Results

### Participant characteristics

The majority of participants completing questionnaires (*n* = 240; 86%) were female (82.5%), White (50%), educated to degree or post-graduate level (44%) and with a mean age of 53 years (range 23–82 years). Almost half (48%) reported fair or poor health, which is linked with higher usage of health services [45], see Table 1. Of those who completed a baseline questionnaire (*n* = 240), 187 also completed a post-intervention questionnaire, 3-month follow up questionnaire, or both (80%).

**Table 1:** Participant characteristics

	Total sample (***n*** = 240)	Participants interview/FG (***n*** = 22)	Yoga Teachers (***n*** = 7)
**Gender**	**n (%)**	**n (%)**	**n (%)**
Female	198 (82.5%)	22 (100%)	5 (72%)
Male	38 (16%)	–	2 (28%)
Missing data	4 (1.5%)	–	–
**Ethnicity**			
White	119 (50%)	12 (55%)	6 (86%)
Mixed race	10 (4%)	–	–
Asian/Asian British	29 (12%)	2 (9%)	–
Black/Black British	28 (12%)	3 (14%)	1 (14%)
Other ethnic group	29 (12%)	2 (9%)	–
Missing data	25 (10%)	3 (14%)	–
**Highest education**			
GCSE’s/O Levels^a^	34 (14%)	2 (99%)	NA
A Levels/college qualification	46 (19%)	3 (14%)	NA
Degree/post-graduate	105 (44%)	11 (50%)	NA
Other e.g. technical	32 (13%)	3 (14%)	NA
Missing data	23 (10%)	1 (5%)	NA
**Number of classes attended**	**Mean (SD)**	**Mean (SD)**	**NA**
	7.7 (2.90)	6.2 (2.17)	NA

^a^General Certificate of Education taken aged 16 in UK

Interview and focus group participants (*n* = 22) were all female (mean age = 56 years; range 28 to 82 years), with half (*n* = 11) educated to degree or post-graduate level. Twelve participants (55%) were White, seven (32%) identified as Black or Ethnic Minority (BAME), and three participants did not report their ethnicity (14%).

### Themes

Three themes were identified during analysis of qualitative data on participants’ experiences of the programme: motivation to attend Yoga4Health, perceived benefits of the programme, and barriers and facilitators to engagement.

#### Motivation to attend Yoga4Health

Baseline questionnaire responses revealed three key motivating factors for attending Yoga4Health: to improve psychological health (*n* = 145; 85%), such as how to manage stress, relax, or improve anxiety or depression; to improve physicality (*n* = 132; 77%), such as improved flexibility, balance, strength, fitness, or increase physical activity; and to improve or maintain their physical health/well-being (*n* = 116; 68%), such as reducing risk factors for disease (e.g. lower blood pressure or cholesterol, lose weight, improve diet, increase energy, or manage aches and pains). Additionally, participants said they wanted to attend to learn about yoga and related techniques (e.g. mindfulness) for self-care or for social reasons such as meeting new people.

Focus groups/interviews revealed why Yoga4Health had been specifically chosen by participants to help with the above issues. Whilst participants expressed a desire to improve their health and well-being, they were conscious of their poor health and lacked confidence to attend standard yoga or exercises classes. A number reported they had done yoga in the past (when they were fitter) or had always been interested in yoga, but felt that it was too hard for them. Knowing that Yoga4Health had been specifically designed for people with health conditions and limited ability and that teachers would be able to understand their needs was important for participants, providing them with the confidence they needed to attend. Confidence in programme suitability was further enhanced when offered through community activities they were already attending.

*“I’d always been fascinated by yoga and I always wanted to try yoga. I always thought my build, my size, my age, I wouldn’t ever be able to. I’d left it too late probably in life to try it anyway. … I was doing Healthy Hearts and they said, we’re doing Yoga4Health, would anybody like to sign up? So I put my hand up.” [focus group, female]*

Other participants said that they had little interest in exercise, but thought yoga might be an enjoyable way to be active. For others, the appeal related to the mind-body approach of yoga, or the belief that Yoga4Health would teach yoga ‘properly’, as opposed to the perceptions of gym-based yoga.

*“I thought it would be good to go to, because I wanted to know more about the breathing. Because you don’t tend to get that in gyms and places, they don’t tell you the theory, and you don’t have time to know how to do the breathing properly.” [Focus group, female]*

#### Perceived benefits of the Yoga4Health programme

Of those who completed the post-intervention questionnaire (*n* = 171), virtually all participants provided responses regarding benefits they had experienced and provided examples of tangible and concrete lifestyle changes they had made. These benefits were echoed in interviews/focus groups and by participants who completed the 3-month follow up questionnaire. Responses regarding benefits were organised into four sub-themes: 1) psychological benefits, 2) physical health benefits, 3) social benefits, and 4) self-management of health and well-being.

##### Psychological benefits

The impact of Yoga4health on mood, stress-management and mental health was widely reported by participants. Many described feeling better in some way, using adjectives such as “awesome”, “more resilient”, “younger”, “peaceful” and “more relaxed”, to articulate this positive feeling. However, the most common psychological benefit reported by participants was an increased ability to cope with stress, which also appeared to contribute to improved mood. In particular, participants described the use of breathing and relaxation techniques learned during Yoga4Health to manage stressful situations in their daily lives, such as the commute to work or dealing with family issues. Some participants explained how they had learned to “take a step back” to recognise and change their reactions to stressors. Furthermore, responses to the three-month follow up questionnaire suggested that these benefits were maintained.

*“I have learnt to breathe properly and to use this mechanism to combat my stress levels … that moment of stress will pass and life goes on.” [female, 60 years, post-intervention questionnaire]*

*“I have continued to feel more relaxed, able to deal with stress better and maintain a positive attitude.” [female, 38 years, follow-up questionnaire]*

Participants’ improved ability to manage stress was also the most prominent benefit discussed by yoga teachers, who gave examples of how participants had applied their new skills in their daily lives with positive impacts ranging from improving family relationships to emotional regulation:

*“All of them pretty much described that they felt more stable and grounded from doing the practice. And most of them described that they had reacted differently to life situations in some shape, way or form whether it was family or friends or something stressful … they're more aware of things that are moving inside them so they feel the anger coming before it’s taken over or they feel the anxiety present before it’s there.” [Yoga teacher 1]*

Some participants described feeling better able to manage existing mental health issues, particularly anxiety. Overall, breathing practices were reported as the most valued component of the course, with many directly attributing the benefits they experienced to these newly acquired techniques. Relaxation exercises were also viewed as valuable for their ease of application in daily life. Participants thus appeared to have developed a range of new skills to support their psychological health, described by one participant as “a very effective personal toolkit”.

*“I think taking away the breathing exercises and just the awareness that you get of your body, taking that away into your everyday life, that aspect of the practice I’m finding very useful.” [P5, female, 69 years, interview]*

*“Mentally, my anxiety levels each day have improved greatly. I always had an awful churned up feeling in my stomach … I have noticed that I no longer have this awful feeling.” [female, 54 years, post-intervention questionnaire]*

However, one participant reported that she felt the breathing exercises exacerbated her anxiety: “It focuses on the breath and for someone like me who suffers panic attacks and rather not think about breathing, it was a quite daunting”. Similarly, a yoga teacher told how one service user had a negative emotional experience during class after becoming aware of difficult emotions in relation to a recent trauma. These comments highlight the divergent experiences of some participants with existing mental health conditions.

Broader psychological impact related to reports of increased self-confidence. Some with complex health needs discussed initial concerns that the classes would be too advanced for them. However, these participants felt happy that they were able to “keep up” with classes, which in turn, increased confidence in themselves and their abilities. A few participants reflected on how this had positively impacted on their “self-image” and identity. For example, one explained how she had not been able to practice yoga in years due to chronic fatigue and so participating in the programme made her feel like her “old self”. Helping participants to become physically active again may therefore bolster their sense of identity with concomitant benefits for mood.

*“I got a lot of confidence back because when I first started doing it, I couldn’t keep up. And I was with people who … like this couple of old nurses who were in their eighties and they were getting on right through this … and I would sadly lie down on the mat and wait, but I just kept going and by the end I was keeping up with everyone doing it so I really, it was very good, and it was, as I say, it was like coming home, it was like all these things that I hadn’t been doing, and I’d stopped doing the yoga. It was brilliant.” [P1, female, 63 years, interview]*

##### Physical health benefits and improved physicality

Participants described a range of physical improvements including increases in flexibility, balance, mobility, posture, strength, and general fitness. Some participants also reported reduced aches, pains, and stiffness. Yoga postures, and the knowledge of which poses help specific issues, were credited with these benefits:

*“When I have a specific pain or injury, I now know which are the best poses and stretches to do.” [female, 52 years, follow-up questionnaire]*

Several participants described improvements to existing health conditions, including respiratory and musculoskeletal conditions, and high blood pressure: “Suddenly my blood pressure has become normal after so many years”. Other physical health benefits included weight loss, improved sleep and increased energy, with some participants directly attributed positive changes in sleep and energy to breathing and mindfulness practices learned during the course. For example, one participant told how she used breathing techniques to help her to go back to sleep upon waking, which improved her overall sleep pattern. Others commented on more chronic sleep problems:

*“My insomnia is almost gone. I have a few nights every now and again when I don’t sleep.” [female, 55 years, follow-up questionnaire]*

Yoga teachers’ accounts of service user’s reports about their experienced benefits further support these findings. For example, yoga teachers told how some participants had experienced improved balance and flexibility, and one yoga teacher reported how some participants had lost weight.

*“I only can pass the feedback that my students give me because we have this gathering time at the end which is very wonderful, it’s just amazing. And every, today for example, just from today, someone mentioning the balance, someone went, “Oh me too!”, so, yes, were commenting on balance, feeling more flexible.” [Yoga teacher 2]*

##### Social benefits

Whilst social benefits were not heavily cited in the post-intervention questionnaire, the interviews revealed that attending the Yoga4Health course created feelings of social connectedness. Participants explained how they preferred the group class to practising at home as they enjoyed being with liked-minded people, with some having formed friendships with their peers. They further described how they found value in hearing other people’s experiences but also sharing their own, both of which were facilitated by the group discussion at the end of classes. This resulted in a “community feel” and served as a “bonding experience” for participants.

Overall, the group element of the Yoga4Health programme appeared to act as vehicle for informational, emotional, and social support. In the baseline questionnaire, only a minority of participants had stated that they hoped to meet others and socialise. However, many participants reported experiencing social benefits, and moreover, appeared to value them greatly.

*“I felt that I was in a very caring environment … And a very warm environment. And I found myself, I’m quite often very cynical and what have you, and I’m quite judgemental as well, and I found myself actually being kind (laughs) and thinking such things as, actually, what a lovely group of people.” [P5, female, 69 years, interview]*

Yoga teachers also observed social changes in the group. For example, they told how the groups became more comfortable with one another at each class, by becoming more talkative and sharing their personal experiences. One yoga teacher described how relationships began to form between the members of the group, as well as between the service users and the teacher:

*“At the beginning the first week everyone is very quiet, no one wants to share much … but then you see that they open up so much and they like to share and so it’s wonderful to see this social bit of this yoga programme, how people get more and more comfortable.” [Yoga teacher 1]*

##### Self-management of health and wellbeing

Participants reported feeling more knowledgeable and in control of their health, with greater confidence and skills to manage their health and wellbeing. For example, one participant described dealing with medical issues that she had previously put off, others discussed making more “positive choices” regarding their health. Health was therefore given greater priority, with some discussing how yoga made them realise the importance of self-care and described how it felt good to do something for themselves, especially for those who spent so much time caring for others, such as children or relatives. The Yoga4Health programme encouraged participants to take “time out” or “make time” for themselves. Consequently, after the course, some participants felt more able and confident to prioritise their own health.

*“What it made me do was reassess my life and how important actually I am, and how I need to give myself something … I reassessed my life basically, and how important it was for me to find time for me to do stuff. To be a bit more autonomous in my own healthcare. Things that I’ve been niggling, like I’ve had a pain in my hip for quite a long time and I’ve never gone to the doctor with it because I can’t face the battle … ” [Focus group 3, female]*

*“The main benefit has been the recognition that I can improve my health and mostly my mental health by using mindfulness, yoga, and meditation to keep me away from the GP.” [female, 57 years, post-intervention questionnaire]*

##### Lifestyle changes

Many participants described changes they had made to their lifestyle as a result of yoga. These lifestyle changes overlapped and interacted with the sub-themes above of improved physical and psychological health, and are indicative of improved self-management and agency over health. For example, nearly a quarter of participants who responded to the post-intervention questionnaire described the positive effects of the Yoga4Health programme on their diet and eating habits, which was further elaborated on within the interviews. Participants described an improved awareness of their bodies as a result of yoga, which in turn, drew their attention to their health and consequently, their diet and related habits.

*“There’s no point in going to yoga and then going and having spaghetti bolognese and three cakes and so it made you more aware of your body and what you were eating and how you were living. I genuinely believe that.” [Focus group 2, female]*

Participants talked about how they became more aware of their eating habits and were able to change to healthier habits by reducing consumption of unhealthy food and increasing fruit and vegetable intake. Since practising yoga was seen as beneficial for health, unhealthy eating was seen to undermine practice.

*“I'm conscious of the food I consume, and am eating more fruit and vegetables in my diet”. [female, 30 years, post intervention questionnaire]*

Other participants described an overall increase in their levels of physical activity, including increases in yoga, walking, or running; where participants were “more active than before” or “more willing to go out jogging”. Some described how they had continued to practice yoga to maintain the physical health benefits experienced:

*“I’ve exercised more and now look forward to it. I’m also walking more”. [female, 46 years, post intervention questionnaire]*

#### Barriers and facilitators to engagement

##### Enjoyment and benefits

How much services users enjoyed the intervention was key to engagement, where users did not enjoy the course or yoga, they were less likely to undertake home practice or continue to practise once the course was completed. However, the majority reported high levels of enjoyment, describing the programme as “enjoyable”, “useful” and even “life-changing”. Enjoyment appeared to be influenced by a number of factors including group dynamics, yoga teacher qualities, inclusivity of the class and practical issues, which are described in more detail below.

*“I have found the course to be a unique and powerful experience. I was really surprised at how much I enjoyed it and looked forward to the sessions.” [female, 55 years, post-intervention questionnaire]*

Experiencing benefits of yoga practice (along with a lack of negative experiences or problems) was key to engagement, particularly participants’ continued practice of yoga once the course had finished. Participants wanted to sustain the improvements to their “mood”, “energy levels”, “body shape” and the “sense of calm” they had experienced.

*“I know how beneficial practicing yoga has been/is for me so that motivates me to continue to practice.” [female, 69 years, follow-up questionnaire]*

In some cases, class venue was found to negatively impact on enjoyment of the programme; several participants complained that the community venues were too cold to practice, especially for those who suffered with pain and related ailments. For example, one service user told how she was in “great pain … which was dreadful” during the classes due to the low temperature of the venue caused by “a window that was open that wouldn’t close” [focus group 2]. This issue deterred some from attending.

##### Suitability of the class

How well the class aligned with participants needs was key to engagement. A few participants felt that the classes were not a ‘good fit’ for them, some reported finding yoga “demanding” or “difficult”. In contrast, one participant found it repetitive and unchallenging (with a few others reporting they felt this way towards the end of the course), others did not enjoy the relaxation at the end of the class. The vast majority, however, felt that the course was for people of their ability, which had been a key motivator for attending the course. The provision of modifications and alternative practices were important enablers, and meant that participants felt able to try even challenging postures, with teachers making “everyone feel included”.

*“The teachers made real good efforts to be very inclusive of everybody and bend over backwards to fit into everybody’s needs” [Focus group, female]*

Timing and location of class were also important. Yoga4Health organisers arranged classes in local community venues and at times that they thought would be most accessible for participants. Nevertheless, a small number of participants could not attend due to scheduling constraints, for example not being able to get time off work to attend. Some recommended having a break in the middle of the course, whilst others suggested a second weekly session or a class recording for those who needed to miss a session. Others, highlighted the sheer time commitment needed from them as challenging in comparison to other methods for managing health and wellbeing:

*“The time commitment compared to popping a pill is a challenge and it is very hard to commit so much time in an already busy life.” [P147, male, aged 53, post-intervention questionnaire]*

##### Practising in a group

Participants highlighted their enjoyment of the group dynamic both for its practical and social encouragement. The vast majority of participants reported a preference for practising yoga in a group compared to practising alone at home. Classes provided several advantages over home practice: participants valued the protected time to practice yoga without distractions, often feeling that they lacked the discipline and time to practise alone at home. Others felt motivated by wanting to ‘keep up’ with others in class. Unlike home practice, the class provided the opportunity to ask questions and have yoga postures corrected whilst receiving encouragement from the teacher. Moreover, participants enjoyed hearing about others’ experiences of yoga, as well as getting to know their peers on a personal level.

*“It was nice to sit around and just hear people’s experiences and share that, yeah. And I have a tendency to want to talk a lot, and I found myself just sitting back and listening, but that was the effect of being in the yoga class. It was interesting to hear people’s different experiences … and to get to know them a little bit better.” [P5, female, 69 years, interview]*

Losing the structure provided by a group class at the end of the programme therefore created challenges for the continuation of yoga practice. In addition to the above factors, some participants felt that without regular classes they simply “fell out of the habit” of practising yoga. However, for others the course kick started a habit which they were able to continue. In addition, the relationships formed within the Yoga4Health course encouraged continuation of practice within their already established social networks. For instance, some participants found new classes to go to together, whilst others practiced yoga at home together.

*“The enthusiasm of class members- we now meet every Tuesday evening and practice yoga together.” [female, 73 years, follow-up questionnaire]*

Our findings highlight that being able to find another suitable class was an important factor for whether or not participants continued to practice once Yoga4Health had finished. Classes needed to be at an accessible location, time and cost, as well as suiting the participants’ ability; there was a perception that local classes would not meet the needs of individuals with complex needs. Recommendations for improvement included additional Yoga4Health sessions and one teacher said they would have liked the ability to provide GPs with feedback regarding participant needs.

*“[I] found it difficult to find a class appropriate to my needs. I have a knee injury. Most classes don’t cater for people with mobility problems.” [female, 65 years, follow-up questionnaire]*

##### Yoga teacher skills and relationship

Having skilled yoga teachers providing Yoga4Health was key to achieving the enjoyment, adaptations, inclusivity and positive social environments described above. Participants valued and praised the skills and qualities of their teacher, describing teachers as “supportive”, “caring”, “dedicated”, “encouraging”, and “knowledgeable”, these qualities also appeared to facilitate adherence to the programme:

*“It was very easy to follow, because the instruction was so good … There are people who can teach and there are people who can’t, and she just had that outgoing, friendly, interested in her class, concerned, good at demonstrations. Yeah, just good teaching is really important, and I think she created that atmosphere of togetherness … While we were there together, I had a feeling of it was quite a unit. And that was teacher creating that.” [P2, female, 82 years, interview].*

Yoga teachers acknowledged that building a relationship with the group was important for both their own and participants’ enjoyment. Rapport was built in various ways, for example, one teacher talked about using humour in the class when participants struggled to achieve certain postures. Communication with participants and being available were also identified as strategies to build the teacher-practitioner relationship. This also helped yoga teachers to adapt the script and/or postures and exercises to suit the individual needs of the group. This skill appeared important in engendering an inclusive approach within classes.

##### Course materials

Participants often reported the course materials (home practice sheets, online home practice videos and course manual) to be a facilitating factor for home practice and subsequent maintenance of practice post-intervention. Irrespective of resource preferences, each offered the benefit of enabling participants to recap what they had learned in class, progress in their yoga postures outside of class, and enabled them to catch up if they missed a class. Although some felt that the quality of presentation of these resources could be improved e.g. sound quality on videos.

*Participant A: Yes, because the video’s OK. And you could pause it and have another look, so that video was really, I think that was the best thing, having those videos.*

*Interviewer: Did anyone else use the videos or manuals in the evening to recap what they learned?*

*Participant B: I choose the manual, just because I wanted to, because I was doing the stretching and that, I wanted to make sure, like, I was doing my warriors properly. [Focus group 1, females]*

## Discussion

This is the first study to report on patient experiences, feasibility and acceptability of a novel multi-component yoga intervention on social prescription for several high-risk patient groups. The biopsychosocial benefits reported by participants and high levels of enjoyment, highlight the potential of this type of programme on social prescription for supporting at-risk groups in the community. Perceived benefits spanned psychological, physical and social domains, with participants continuing to report benefits at 3 months post intervention. Improved self-management skills, along with a greater awareness and prioritisation of self-care were also notable, with a number of participants reporting positive lifestyle changes after attending classes. Whilst motivation to initially attend often centred around a desire to improve physical or mental health, unanticipated benefits emerged for participants, such as enjoyment and a sense of social connection, which further supported ongoing attendance and practice. The qualities of the yoga teacher, access to a suitable class, and other practical considerations were also important for ongoing engagement and practice (during and after the intervention). Home practice was supported by Yoga4Health course materials, as well as learning practical techniques (such as breathing exercises) that could be easily applied in everyday life with immediate benefit. A key challenge was continuation of practice once the support and structure of the group classes had finished, highlighting the importance of finding suitable classes for these patient groups in terms of cost, location, timing, and ability level.

The improved mental and physical health reported by study participants are consistent with the significant literature citing these benefits for our patient groups [10, 13, 22, 46]. Developing self-regulation skills enabled better management of stress and improved mood, with many discussing the importance of breathing practices (pranayama) to manage emotions and stressful situations. This aligns with previous studies demonstrating the effectiveness of breathing techniques for stress management and mood enhancement, potentially through regulation of the autonomic nervous system [30, 47–49]. Such practices are also likely to be important in the longer-term maintenance of patient-reported benefits. Physical health benefits were broad ranging, including balance and mobility, general fitness and weight loss. Some participants also reported improvements to secondary health conditions, such as high blood pressure, which requires more systematic investigation but is consistent with previous research [13, 48].

The social benefits reported in the current study are supported by a small but growing number of studies that have investigated the effects of yoga on social connection, in both the general population and patient groups. Practising yoga with a group can provide opportunities to meet (potentially like-minded) others and engender a sense of social connectedness [12, 27, 28, 50], this may be particularly important for groups with the same health conditions practising together [31]. Indeed, the group element of the Yoga4Health programme appeared to act as vehicle for informational, emotional and social support. Specific activities to support the social cohesion of a yoga group (e.g. time for discussion), as delivered by Yoga4Health, are likely to maximise these social benefits, the importance of which may be currently underestimated. Given the positive relationship between social interaction and mental health (as well as social isolation with mortality risk) [51], group yoga may provide an opportunity to increase social capital in high-risk groups – future research should seek to examine this aspect of yoga more extensively. It is important to note that whilst psychological, physical and social benefits have been separated in our reporting, these aspects are in fact inextricably interlinked, for example, improved physical functioning can lead to better mental health, as acknowledged by our participants and research [52].

Of key importance for this study was the finding that participants reported improved self-management skills including the knowledge, skills and confidence needed to manage one’s health, suggesting improved patient activation among our participants [53]. Patient activation has been identified by the NHS as an important factor in supporting people to manage their own health and is encouraging interventions that can promote it [54]. The multi-component nature of yoga suggests it could be an ally for improving patient activation; our qualitative data identified a number of avenues which support patient activation, in line with previous yoga studies, such as improved confidence [31], personal responsibility for health [55] and a sense of mastery of new skills and knowledge [36]. In addition, yoga can increase awareness of the body and what it needs to be healthy, prompting positive health choices [56]. This was reported by participants in our study and in similar populations, where those with CVD or diabetes, began to lose their desire to eat unhealthy food and increase their physical activity [16, 31]. Taken together these findings suggest that yoga may be a useful intervention for stakeholders looking to promote patient self-management and activation.

For those promoting yoga interventions, it is worth noting that studies of yoga and complementary therapies have demonstrated that reasons for continuing practice often differ from, or expand on, reasons for initiating it [6, 31, 57]. In our study, motivation to initially attend often centred around a desire to improve physical or mental health. As participants progressed with classes other benefits emerged, such as enjoyment, group support and boosts to self-confidence, which motivated ongoing attendance and practice. Similarly, Thind et al., (2019) reported that the initial motivation for their participants with diabetes to attend a yoga intervention was to improve their physical health, yet increases in self-confidence, feelings of relaxation and accountability to the group were reasons for continuing practice [31]. It is likely that those who have not tried yoga previously are unaware of the wider benefits these types of intervention can bring, which may reduce uptake particularly in more vulnerable or marginalised groups [35, 36].

Our findings showed that Yoga4Health appeared to be acceptable to the majority of patients; the ‘enjoyability’ of yoga was important here, and this finding is reflected by other research [16, 58, 59]. Enjoyment is likely to be key to sustaining engagement and benefits of an intervention by increasing intrinsic motivation [60, 61]. Yoga may offer a form of physical activity that is more enjoyable and accessible to many than more traditional forms of exercise, and future research should seek to understand why this is the case, for example is the mind-body approach appealing? Additionally, having a yoga programme designed specifically for long-term conditions was important both for initial attendance and adherence. The skill and qualities of the yoga teacher were important facilitators for positive and inclusive experiences, with participants highly valuing teachers for being supportive, caring, dedicated, encouraging and knowledgeable. The importance of the teacher for intervention acceptability, adherence and enjoyment has been widely reported in previous studies [30, 36, 55].

Participants reported very few negative reactions or problems related to practice, however, it is important to note that a small number of participants reported triggering of anxiety-related symptoms. The complex needs and abilities of this patient group suggest the importance of highly trained yoga teachers with experience of working with patient populations. Indeed, the Yoga4Health programme was designed by yoga therapists, and delivered by experienced teachers who underwent specific training and received ongoing support. In acknowledgement of the importance of the teacher’s role in managing patients’ complex needs, the Yoga4Health teacher training course has now been increased from 35 to 60 h and has been accredited by the Personalised Care Institute.

Finding time for a 2-h class did act as a barrier to attendance for some. Time barriers are common for lifestyle interventions [62], nevertheless Yoga4Health minimised other key attendance barriers such as cost [62] by operating through a social prescribing route, and providing classes at largely accessible times and venues. Recommendations for improving Yoga4Health included ensuring class venues were appropriate, improving the presentation quality of home practice materials, providing more sessions for those who wished to continue, more variability in sessions towards the end of the programme, having increased flexibility of sessions for those who missed a class, and providing a mechanism to allow teachers to provide feedback on participants to GPs or refer on in other ways.

For some participants attending Yoga4Health kickstarted a practice or ‘habit’ that they were able to continue after the intervention ended: some found new classes to attend, others used the social connections they had formed and practised at home or attended new classes together. Whilst some participants used their home practice materials to continue their practice, our findings indicated that many participants benefitted from attending a class to develop and support regular ongoing practice. As well as the aforementioned benefits of classes, attending a class also meant that there was protected time for practice, supporting habit formation [63]. When some participants were unable to find a suitable class (due to cost, location, time, ability), practice was discontinued, a key barrier found in other studies [36, 64]. Supporting intervention participants on an individual basis to find a suitable class could support the continuation of yoga practice and the benefits it brings, for those who wish to continue. It was also evident that simple practices that could be implemented ‘off the mat’, particularly breathing techniques, were important to supporting daily practice, which has been found by other studies [48, 55, 65, 66].

### Strengths and limitations

The diverse sample and triangulation of three qualitative methods plus both participant’s and yoga teacher’s perspectives are strengths of the current study [67, 68]. Additionally, the inclusion of a 3-month follow-up questionnaire provided valuable information about continued benefits and practice. However, there are limitations to consider. For example, whilst efforts were made to recruit for interview and focus group and collect follow-up questionnaires from those who had not attended all their sessions, the final participant sample may have had more favourable views about the programme. In addition, surveying those who chose not to participate in the intervention would have provided additional information regarding barriers to attendance. Participants were not specifically asked about individual elements of Yoga4Health classes (e.g. asana, relaxation), future studies may wish to delve deeper into participant experiences of each element, particularly the group discussion which is a unique feature of Yoga4Health. While our sample was ethnically diverse, it was predominantly female, however, women are much more likely to practise yoga in the West [6, 10]. This suggests that yoga as an intervention may be less acceptable to men with long-term conditions, which should be explored with future research. A controlled study of Yoga4Health is now warranted, to examine benefits more systematically.

## Conclusions

Yoga4Health was an acceptable intervention to patients, which was perceived to bring a wide range of biopsychosocial improvements, including physical and mental health benefits, social connectedness, patient activation and positive lifestyle changes. Taken together these factors suggest that yoga is an appropriate intervention to offer on social prescription, given that social prescribing aims to support patients (particularly those with long-term conditions) in a holistic way. Quantitative studies including clinical trials, should now be used to explore this intervention further, our findings can be used to guide the design of such research. Future research may also wish to examine this model of delivering yoga as a health intervention for other conditions, as well as how to support patients to continue their practice in order to promote ongoing self-management of health and wellbeing.

## Supplementary Information

**Additional file 1.**

**Additional file 2.**

**Additional file 3.**

**Additional file 4.**

## Data Availability

The datasets generated and/or analysed during the current study are not publicly available since complete transcripts of interviews and focus groups potentially allow for identification of individuals, but are available from the corresponding author on reasonable request.

## Abbreviation

NHS: National Health Service
