# Supplementary material for: ‘Joining a group was inspiring’: a qualitative study of service users’ experiences of yoga on social prescription
Source: BMC Complement Med Ther. 2022 Mar 14;22:67. doi: 10.1186/s12906-022-03514-3 (PMC8922896; doi:10.1186/s12906-022-03514-3)
Supplement: Supplementary file 1 — Additional file 1. [file 12906_2022_3514_MOESM1_ESM.docx]

**Additional file 1 – Intervention exclusion criteria**

Exclusion criteria included:

- severe/acute depression and anxiety;
- post-traumatic stress disorder;
- recent psychotic episode (the last 5 years) or schizophrenia;
- bipolar disorder;
- pregnancy;
- recovering from substance misuse;
- recovering from an eating disorder;
- diagnoses of anti-social behaviour issues;
- ME/CFS;
- osteoporosis, osteopenia, fibromyalgia;
- severe or acute muscular-skeletal issues;
- COPD;
- acute asthma;
- kidney failure;
- Parkinson’s disease;
- cognitive impairment.
