# Supplementary material for: ‘Joining a group was inspiring’: a qualitative study of service users’ experiences of yoga on social prescription
Source: BMC Complement Med Ther. 2022 Mar 14;22:67. doi: 10.1186/s12906-022-03514-3 (PMC8922896; doi:10.1186/s12906-022-03514-3)
Supplement: Supplementary file 2 — Additional file 2. [file 12906_2022_3514_MOESM2_ESM.docx]

**Additional file 2 - Yoga4Health participant interview schedule**

Opening question

- What made you decide to attend the yoga course? What did you hope to get out of it?
- Why did you choose to attend this particular yoga class over another?

**Topic 1: Outcomes**

Benefits

- Were your expectations of the course met?
- What was the main thing you got from the course?

Specific outcomes?

- Do you think the yoga course has had an effect on physical heath? If so, how?
- Do you think the yoga course has had an effect on your well-being? If so, how?
- Do you think the yoga course has had an effect on your social life? If so, how?
- Are you doing anything differently in your life after attending your yoga course?

Disadvantages

- Did you experience any negative consequences as a result of taking part in yoga course? If so, what were they? (prompt, felt ill after a class, had an injury related to doing yoga)

Home practice (outcomes and process)

- How did you find the practising at home between each class? (prompts: how often did you manage to do, what aspects did you tend to practise most frequently/find most useful)
- During the yoga course, which out of the course materials did you use or find useful? (e.g. videos, manual, home practise sheets)
- Have you continued/do you think you will continue to practice yoga now that the course has finished? If so, why? What aspects of the course have you continued with? (e.g. videos, manuals etc)
- Was there anything that made it difficult to continue to practise yoga after the sessions finished?
- Was there anything that helped you to continue to practise yoga after the sessions finished?

**Topic 2: Process**

Perceived ease of use

- How did you find practising yoga?
- How did you find the yoga course? Was it easy or difficult to follow/understand?

Facilitators

- What did you like about the yoga classes? Why?
- Was there anything that made the yoga classes easier for you? How so?

Barriers

- Was there anything that you didn’t like about the yoga course?
- Was there anything that made the yoga classes more difficult for you? How so?
- Was there anything that prevented you from going to a class?

Perceived usefulness

- What were the most useful parts of the course? Why?
- What were the least useful parts of the course? Why?

Future development

- Do you think the course can be been improved in any way? If so, how?

Closing question

- Overall, how did you find being involved in the study?
- Is there anything you’d like to add about the yoga and the Yoga4Health course that we’ve not already covered?
