# Supplementary material for: ‘Joining a group was inspiring’: a qualitative study of service users’ experiences of yoga on social prescription
Source: BMC Complement Med Ther. 2022 Mar 14;22:67. doi: 10.1186/s12906-022-03514-3 (PMC8922896; doi:10.1186/s12906-022-03514-3)
Supplement: Supplementary file 3 — Additional file 3. [file 12906_2022_3514_MOESM3_ESM.docx]

**Additional file 3 - Yoga4Health participant focus group schedule**

1. Introduction: Brief information on the Yoga4Health research study. Reminder of the aims of the study and what topics are expected to be covered in the focus group (5 mins).
2. Brief group introductions: names and venue of yoga class (2 mins).
3. Information on ground rules: treating others’ comments confidentially, listening to what other group members have to say, and not talking over other participants. Participants will be given the opportunity to add any other group rules that they feel are appropriate (5 mins).

**Topic 1: Motivation for joining the course**

- What made you decide to attend the yoga course? (Prompt: What did you hope to get out of it?)

**Topic 2: Outcomes**

- Did you experience any benefits as a result of taking part in the yoga course? (Prompt: Physical heath, well-being, social life; Are you doing anything differently in your life since attending the course?)
- Did you experience any negative consequences as a result of taking part in yoga course? (Prompt: negative emotions, pain etc)

**Topic 3: Process**

- How did you find the yoga course? (Prompt: easy or difficult?)
- What did you like most about the yoga course? Why? (Prompt: Was there anything that made the yoga course easier for you? Were there aspects that you found most useful?
- Were there any challenges on the course? (Prompt: Was there anything that you disliked? Was there anything that made the yoga course more difficult for you? Were there aspects that you found least useful?)
- How did you find the home practice? (Prompt: What aspects did you find useful/not useful? Easy/difficult? Benefits?)

**Closing question**

- Have you continued/do you think you will continue to practice yoga now that the course has finished? Why
