# Supplementary material for: ‘Joining a group was inspiring’: a qualitative study of service users’ experiences of yoga on social prescription
Source: BMC Complement Med Ther. 2022 Mar 14;22:67. doi: 10.1186/s12906-022-03514-3 (PMC8922896; doi:10.1186/s12906-022-03514-3)
Supplement: Supplementary file 4 — Additional file 4. [file 12906_2022_3514_MOESM4_ESM.docx]

**Additional file 4 - Yoga4Health stakeholder interview schedule, Yoga teachers**

Opening question

- What made you decide to get involved in teaching the Yoga4Health course?

**Topic 1: Process**

Perceived ease of use

- How did you find the delivering the classes? Were they easy or difficult to provide?

Barriers

- What were the challenges delivering the Yoga4Health course for you?

Facilitators

- Was there anything that helped the classes to run smoothly?
- What aspects of the course worked well/less well? (e.g. different aspects of the course, such as breathing, asana, psychoeducation/discussion, and duration of the course and classes)

Type of person

- What types of people to you think attended the course?
- What type of person do you think the course would benefit most from the course?
- Can you tell us about those participants who dropped out of the course – what were the main reasons for this (if known)?

**Topic 2: Outcomes**

Benefits

- In your opinion, do you think the participants experienced any benefits as a result of taking part in the yoga course? (prompt: did participant report any benefits or did you observe any changes in them?)

Perceived usefulness

- What do you think were the most useful aspects of the course for participants? Why?
- What do you think were the least useful aspects of the course for participants? Why?

Disadvantages

- Do you think the participants experienced any problems as a result of taking part in the yoga course? (prompt: injuries)
- Were there any issues that were brought up by the participants?

Future development

- Do you think the course can be been improved in any way? If so, how?
- What advice would you give to other teachers delivering the course
- Is there anything you’d like to add about the Yoga4Health course that we’ve not already covered?

Closing question

- Overall, how did you find being involved in the study?
